# From Bulk to Spatially Resolved Single-Cell Omics: Shaping Future Prognostic and Predictive Stratification in Head and Neck Squamous Cell Carcinoma

**DOI:** 10.3390/cancers18142223

**Published:** 2026-07-10

**Authors:** Simonetta Ausoni, Alessandra Casarin, Giuseppe Azzarello

**Affiliations:** 1 Department of Biomedical Sciences, University of Padova, Via Ugo Bassi 58b, 35131 Padova, Italy; 2 Department of Oncology, Local Health Unit 3 Serenissima, Mirano Hospital, Via don Giacobbe Sartor 4, 30035 Venezia, Italy

**Keywords:** HNSCC, bulk transcriptomics, single cell transcriptomics, spatial transcriptomics, patient stratification, locally advanced HNSCC, recurrent/metastatic HNSCC, precision medicine

## Abstract

Head and neck squamous cell carcinoma (HNSCC) is characterized by marked intratumoral heterogeneity and complex tumor–immune–stromal interactions, which shape therapeutic response and clinical outcome. Despite extensive transcriptomic efforts, bulk RNA sequencing has faced significant limitations, often failing to generate robust prognostic or predictive biomarkers, highlighting the need for approaches capable of resolving the cellular and spatial complexity of the tumor ecosystem. Single-cell RNA sequencing (scRNA-seq) and spatial transcriptomics (ST) have refined our understanding of HNSCC biology by enabling high-resolution mapping of malignant, stem-like, immune, and stromal compartments. Three major spatial domains have been defined in HNSCC: tumor core (TC), tumor invasion front (TIF), and leading edge (LE). Each ecosystem exhibits distinct cellular programs that promote immune evasion, tumor dissemination, and therapy resistance, particularly in high-risk clinical settings. In this Review, we integrate recent single-cell and spatial studies and propose a translational framework linking ecosystem architecture with clinical stratification across resectable locally advanced (r-LAD), unresectable locally advanced (u-LAD), and recurrent/metastatic (R/M) disease. We further discuss how spatially resolved transcriptomic approaches may support biomarker discovery and hypothesis generation for risk stratification and trial design, while emphasizing that clinical implementation remains limited by cohort size, methodological heterogeneity, and the need for large-scale prospective validation. Finally, we outline key methodological and translational challenges that must be addressed before these technologies can reliably inform precision oncology and decision-making in HNSCC.

## Simple Summary

Head and neck squamous cell carcinoma is a complex disease composed of different cell types that interact and influence how cancer grows and responds to treatment. Traditional transcriptomics approaches analyze tumors in bulk and may overlook important differences between individual cells and distinct tumor regions. Emerging single-cell and spatial transcriptomic technologies allow researchers to study tumors in much greater detail, revealing how different cell populations are organized and communicate within the tumor microenvironment. These advances are providing new insights into why some tumors are more aggressive, spread more easily, or become resistant to therapy. By capturing cancer complexity at higher resolution, these technologies provide a framework for the discovery of more powerful biomarkers and for the generation of hypotheses relevant to patient stratification. Although further validation in large, well-annotated patient cohorts is needed before widespread clinical use, these technologies are establishing an important foundation for the development of more individualized therapeutic strategies in this disease.

## 1. Introduction

Head and neck squamous cell carcinoma (HNSCC) represents a major global health burden, characterized by aggressive clinical behavior and limited therapeutic progress. HNSCC accounts for more than 900,000 new cases annually worldwide, 480,000 deaths annually [1], and is associated with high rates of locoregional recurrence, nodal metastases, and therapy resistance. It arises from the epithelial lining of the upper aerodigestive tract, including the oral cavity, pharynx, larynx, and sinonasal regions. The molecular pathogenesis of HNSCC reflects a complex interplay of distinct embryological origins, genetic alterations, epigenetic modifications, and environmental exposures [2,3,4,5]. Two major etiological pathways contribute to the disease: carcinogen-driven tumors, primarily linked to tobacco and alcohol exposure, and human papillomavirus (HPV)-associated tumors, which predominantly arise in the oropharynx [5,6,7].

Despite advances in multimodal treatment, clinical management of HNSCC remains constrained by limited biological stratification. Current clinical treatment largely relies on anatomical staging and a restricted set of molecular biomarkers, including HPV status and programmed death-ligand 1 (PD-L1) expression [8,9,10]. However, these parameters fail to capture the biological complexity of the disease, resulting in marked variability in treatment response and clinical outcomes. Therapeutic efficacy remains suboptimal, with only modest improvements in overall survival, particularly in patients with unresectable locally advanced disease (u-LAD) and recurrent or metastatic (R/M) tumors. Cisplatin-based chemotherapy remains the standard of care for u-LAD HNSCC, yet it provides limited survival benefit and is associated with high rates of locoregional recurrence, despite the introduction of novel agents and alternative treatment schedules [11,12,13]. Similarly, the incorporation of targeted therapies, such as cetuximab, and immune checkpoint inhibitors (ICIs) into standard regimens has produced only limited benefits, often restricted to selected subgroups of patients [14,15,16,17]. In the R/M setting, prognosis remains poor, with median overall survival generally below one year with first-line platinum-based chemotherapy, only modestly improved by the addition of cetuximab [18]. Although ICIs have improved outcomes in subsets of patients, durable responses remain limited to a minority of cases [19].

These clinical limitations underscore the profound biological heterogeneity of HNSCC and the urgent need for more refined molecular stratification. The identification of robust prognostic and predictive biomarkers, therefore, remains a priority in translational research.

High-throughput transcriptomics profiling has emerged as a powerful strategy to dissect this complexity and advance precision oncology in HNSCC. Since 2011, when the first comprehensive whole-exome sequencing analysis of 74 HNSCC tumors was reported [20], numerous studies have expanded our understanding of the genomic drivers of this disease. These include recurrent alterations in TP53, CDKN2A, NOTCH1, PIK3CA, and EGFR in HPV-negative tumors, as well as the distinct molecular features of HPV-positive disease, driven by the viral oncogenes E6 and E7, alongside alterations shared with HPV-negative tumors [7,21]. A major advance was achieved with the integration of transcriptomic data into large-scale genomic consortia. In 2015, The Cancer Genome Atlas [2] reported the first comprehensive RNA-sequencing analysis of HNSCC, integrating bulk RNA sequencing, whole-exome sequencing, and DNA methylation profiling across 279 tumors. Although transformative, this approach relied on bulk tumor analysis, which captures averaged transcriptional signals across heterogeneous cell populations, and therefore fails to resolve spatial organization and cell-type-specific transcriptional programs, which are key determinants of metastatic dissemination and immune evasion in HNSCC [22,23,24,25].

The advent of single-cell and spatial transcriptomic technologies has begun to overcome these limitations by enabling high-resolution mapping of tumor compartments. The application of single-cell RNA sequencing (scRNA-seq) to HNSCC was pioneered by Sidharth Puram and colleagues [26], who analyzed approximately 6000 cells from primary tumors and lymph node metastases, revealing previously unrecognized cellular heterogeneity. More recently, spatial transcriptomics (ST) has further extended these insights [27,28,29], enabling investigation of tumor architecture and cellular interactions within their native spatial context. These studies have contributed to the emerging concept of the tumor ecosystem, in which malignant cells dynamically interact with stromal, immune, and vascular compartments to shape tumor progression and therapeutic response [30,31].

In a disease driven by cellular plasticity and dynamic cell–cell interactions, these technologies provide new opportunities to redefine HNSCC biology. This Review critically examines the potential of transcriptomics to address key clinical challenges of this disease. We first outline the methodological and conceptual foundations of omics approaches and then delineate the complex cellular heterogeneity that characterizes the HNSCC ecosystem, with particular emphasis on tumor cells, cancer stem cells, and the stromal and immune microenvironment. We subsequently discuss the clinical impact of omics in HNSCC, highlighting emerging strategies that have informed early-phase clinical investigation. Finally, we propose a conceptual framework for patient stratification, rational therapeutic selection, and the design of innovative clinical trials based on omics-derived biomarkers.

## 2. Search Strategy and Data Selection Criteria for Clinical Studies

This Review was conceived as a narrative, hypothesis-driven synthesis of recent advances in bulk, single-cell, and ST in HNSCC, rather than as a systematic review. Literature searches were performed using PubMed/MEDLINE, Web of Science, and ClinicalTrials.gov, complemented by manual screening of references from relevant original articles and reviews. Searches were primarily conducted between January 2022 and February 2026 and included publications available up to February 2026. Representative search terms included combinations of: (“head and neck squamous cell carcinoma” OR HNSCC) AND (“single-cell RNA sequencing” OR scRNA-seq OR spatial transcriptomics OR spatial omics OR tumor microenvironment OR immunotherapy OR epithelial–mesenchymal transition OR EMT OR stemness OR clinical trial). Clinical studies were not selected to provide a comprehensive inventory of ongoing therapeutic trials in HNSCC. Instead, studies were included according to the following criteria: evaluation of therapeutic strategies targeting biological pathways discussed in this Review; availability of clinical data beyond the preclinical stage; and relevance for illustrating how omics-derived results are being translated into biomarker-guided therapeutic strategies. Accordingly, Table 1 should be considered as a representative overview of mechanism-based therapeutic approaches, rather than an exhaustive catalogue of active clinical trials.

## 3. Methodological and Conceptual Insights: Bulk, Single-Cell, and Spatial Transcriptomics

**Figure 1:**
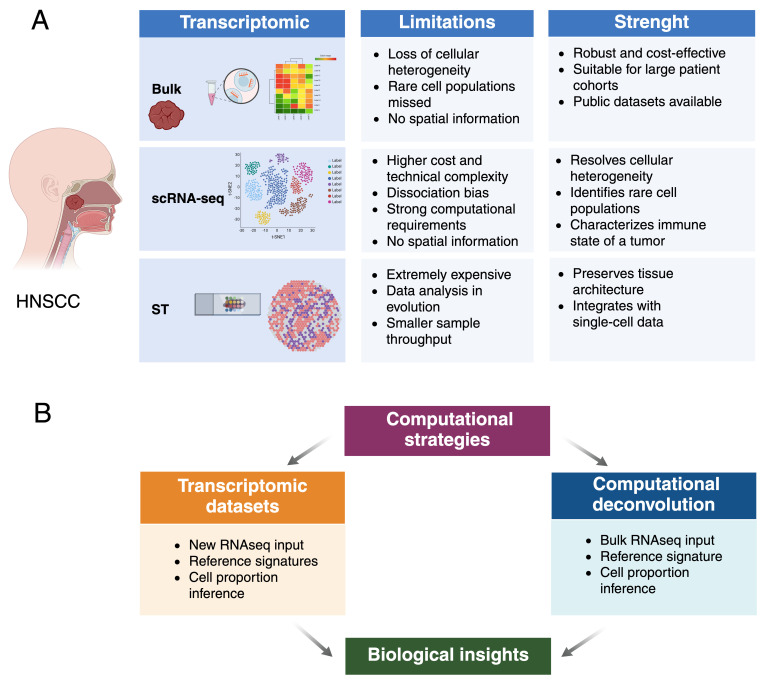
Conceptual framework of transcriptomics and computational approaches in HNSCC. (**A**) Schematic overview of bulk, single-cell, and spatial transcriptomic approaches, highlighting their respective strengths and limitations. (**B**) Transcriptomic data and biological insights can be derived from newly profiled patient-derived tumor samples or retrieved from publicly available repositories. In parallel, computational deconvolution of bulk RNA sequencing data, based on reference gene expression signatures, enables estimation of the relative abundance of cellular populations within tumors. This figure was created with BioRender.com.

Transcriptomic technologies provide a conceptual and methodological framework to link genetic alterations with functional cellular states in cancer. Omics have profoundly reshaped cancer research. While genomics has delineated the mutational landscape and proteomics has provided insight into functional alterations, transcriptomics represents a critical intermediary between genotype and phenotype. In parallel, epigenomics has expanded our understanding of gene regulation beyond genetic alterations. A comprehensive methodological description of these approaches is beyond the scope of this Review; however, we outline here the key conceptual features, strengths, and limitations of the three principal transcriptomics strategies currently applied in oncology (Figure 1A).

Bulk transcriptomics remains the most established and clinically accessible approach, although it is inherently limited by signal averaging. Bulk RNA-seq provides a global overview of gene expression patterns. Over the past two decades, this approach has become the most adopted and cost-effective in cancer research and continues to represent the most accessible for clinical applications [32]. Technically, bulk RNA-seq relies on standardized laboratory workflows, including total RNA isolation, cDNA synthesis, library preparation, and high-throughput sequencing. However, downstream bioinformatic analyses remain computationally demanding, requiring sequence alignment to a reference genome and statistical modeling to identify differentially expressed genes across samples [33]. In HNSCC, the major problem of bulk RNA-seq lies in the inability to resolve the biological complexity of the tumor ecosystem. Although bulk RNA-seq has generated extensive datasets describing gene expression patterns, its limitations obscure intra-tumoral heterogeneity and mask rare—but clinically relevant—cellular subpopulations. Furthermore, bulk transcriptomics lacks spatial resolution and provides only static snapshots, thereby failing to capture the dynamic and spatially organized nature of HNSCC ecosystems.

scRNA-seq overcomes many of these limitations by resolving transcriptional heterogeneity at single-cell resolution [34]. The workflow involves several key steps, beginning with the isolation of single cells, using approaches such as fluorescence-activated cell sorting (FACS), magnetic-activated cell sorting (MACS), or microfluidic droplet-based systems. In the latter, single cells are encapsulated together with barcoded beads carrying unique molecular identifiers, thereby enabling each transcript to be traced back to its cell of origin [35,36]. Following cell isolation, RNA is extracted, reverse-transcribed into cDNA, and processed into sequencing libraries for high-throughput analysis using platforms such as Illumina or Chromium systems. The major strength of scRNA-seq extends beyond the identification of discrete cell populations, enabling the reconstruction of functional cellular states and dynamic trajectories [37,38]. However, these datasets require advanced computational analysis pipelines implemented through frameworks such as Seurat, Scanpy, and Monocle, which support dimensionality reduction, clustering, and differential gene expression analyses across cellular populations [39,40]. These approaches have revealed transcriptionally distinct cellular states and rare subpopulations that remain undetectable using bulk transcriptomic analyses. Beyond static transcriptional profiling, scRNA-seq data can also be used to infer functional behaviors, including pathway activity and cell–cell communication, through computational frameworks such as GSVA, AUCell and PROGENy. In HNSCC, these analyses have facilitated the identification of transitions between proliferative, invasive, and therapy-resistant cellular states. Integration with RNA velocity models, including those implemented in scVelo, enables inference of the directionality of cellular dynamics, thereby providing insight into future cell states and tumor evolution [25].

ST further extends this analysis by integrating molecular information with tissue architecture. By preserving spatial context, ST enables the mapping of gene expression profiles to their original anatomical locations within tissue sections [41,42]. Platforms such as 10× Genomics Visium, Slide-seq, and Stereo-seq provide this information through spatial barcoding that associates transcripts with spatial coordinates [43]. This approach is particularly relevant in HNSCC, where tumor interactions with stroma and immune cells are spatially organized.

Despite their potential, single-cell and spatial approaches are not yet fully scalable to routine clinical practice. Current limitations include high costs, technical complexity, and the need for substantial computational resources and advanced statistical modeling [44]. Moreover, single-cell transcriptomic datasets are characterized by extreme sparsity, high dimensionality, and substantial technical variability, with dropout events and batch effects often obscuring biologically meaningful signals and limiting comparisons across studies. The rapid proliferation of analytical tools and computational pipelines has further generated a fragmented ecosystem, with limited standardization and benchmarking across platforms [45]. In ST, additional challenges include labor-intensive sample preparation and variable RNA capture efficiency, which may hinder the detection of low-abundance transcripts. Rapid technological progress is expected to facilitate the integration of these approaches into clinical workflows over the next decade.

Computational integration and secondary analyses are increasingly central to extracting clinically relevant information from transcriptomic datasets (Figure 1B). In HNSCC, studies may involve either the generation of novel patient-derived datasets or the reanalysis of publicly available data using advanced computational frameworks, often revealing biologically meaningful patterns not identified in the original analyses. In this context, computational deconvolution methods have expanded the utility of bulk RNA-seq by estimating the relative abundance of distinct cellular populations based on reference gene expression signatures. However, these approaches require careful interpretation, as public datasets may not fully capture the biological diversity of HNSCC subtypes and the heterogeneity of real-world patient populations. Similarly, deconvolution approaches may under-represent rare cellular subsets or subtle transcriptional differences with potential clinical relevance. By contrast, scRNA-seq and ST provide complementary and more direct strategies to reconstruct tumor complexity and dynamics. Through trajectory inference methods, these technologies enable modeling of cell-state transitions and the reconstruction of pseudo-temporal trajectories, thereby providing a dynamic view of tumor progression [25].

## 4. Malignant Cell Heterogeneity

**Figure 2:**
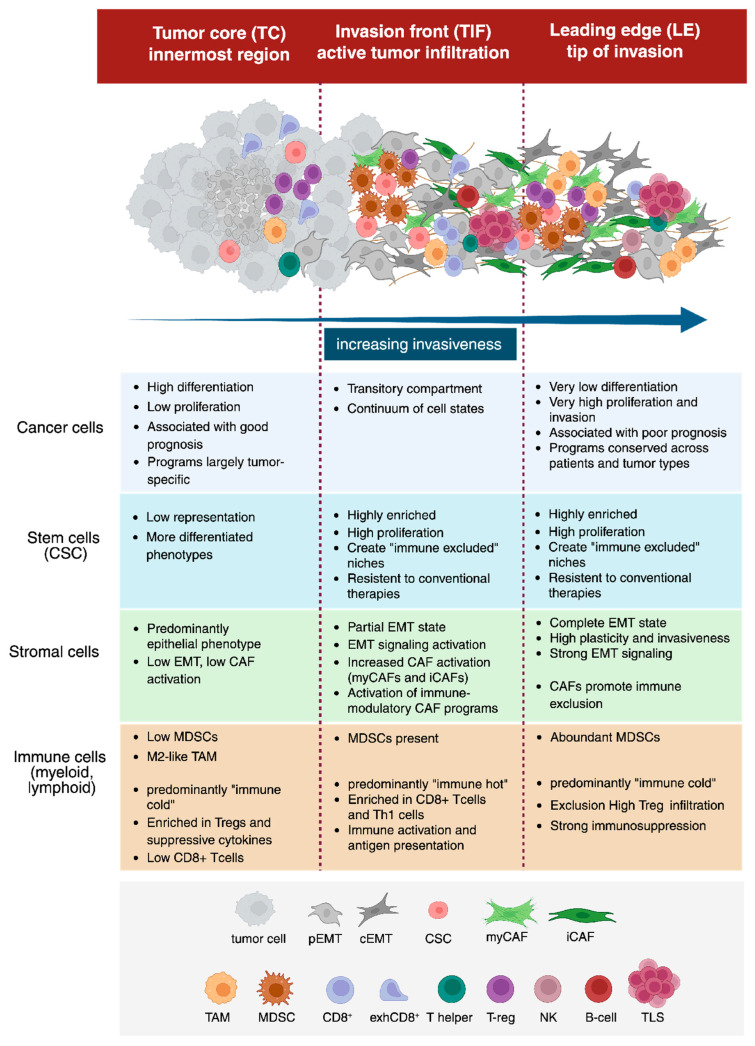
Spatial organization and cellular composition of HNSCC. Three spatial domains are identified: TC, TIF, and LE. Each compartment contains heterogeneous populations of cancer cells, CSCs, stromal cells, and immune cells. Distinct cellular programs within each compartment shape specific biological properties. The figure does not depict the full spectrum of immune and stromal cells detectable in HNSCC but rather focuses on those implicated in tumor evolution and prognosis based on scRNA-seq and/or ST analyses. This figure was created with BioRender.com.

The spatial organization of HNSCC has been described in multiple studies as comprising three major, partially overlapping domains with distinct cellular composition: the Tumor Core (TC), the Tumor Invasion Front (TIF), and the Leading Edge (LE). The TC corresponds to the innermost part of the tumor mass, the TIF marks the region of active tumor infiltration, and the LE represents the most distal extent of invasive growth into surrounding tissue. In this Review, we adopt this terminology; however, in other studies, the term LE may be used to encompass both the TIF and LE. All three regions contain malignant, immune, and stromal cells but differ in their intrinsic properties and relative proportions (Figure 2). TC, TIF and LE should be viewed as dynamic ecosystems rather than fixed anatomical compartments, each defined by cellular states and networks of autocrine and paracrine ligand–receptor interactions. Recent ST analyses suggest that LE is a self-reinforcing invasive ecosystem, sustained by local autocrine signals involving TGF-β1, ICAM1, and Tenascin-C, together with ligand–receptor interactions among malignant, stromal, and immune cells [46]. The extent to which this three-domain organization applies across HNSCC remains uncertain and is likely influenced by molecular and clinical heterogeneity of the disease, including differences in mutational landscapes, signaling networks, and evolutionary trajectories among oral cavity, laryngeal, hypopharyngeal, and HPV-positive oropharyngeal cancers [47,48,49,50,51]. This variability is further compounded by the fact that most currently available scRNA-seq and ST datasets derive from anatomically heterogeneous cohorts. Future studies profiling individual HNSCC subsites will be essential to determine whether the TC/TIF/LE framework represents a conserved map or instead reflects distinct spatial architectures across anatomical and molecular contexts.

Malignant epithelial cells are the primary source of heterogeneity in HNSCC. Clinical omics studies show marked inter- and intra-tumoral heterogeneity and plasticity in tumor cells, whereas immune and stromal compartments display more conserved transcriptional programs across patients. Early single-cell profiling of primary tumors and matched lymph node metastases from 18 HNSCC patients (~6000 cells) first highlighted this distinction [26]. Other studies with expanded datasets demonstrated that tumor cells segregate into transcriptional programs linked to specific anatomical compartments. Keratinocyte-like populations in the TC are enriched for differentiation-associated genes and correlate with favorable clinical outcomes, whereas proliferative cells at the LE display invasive signatures associated with poor prognosis [52,53]. A central feature of the invasive phenotype is the presence of epithelial cells undergoing epithelial–mesenchymal transition (EMT), which contributes to metastatic dissemination and therapy resistance. Cells with EMT-associated programs are particularly enriched at the LE and within the TIF, where they orchestrate local invasion and metastatic dissemination. Recent high-plex ST further supports this concept by showing that tumor buds, identified as clusters of cells detaching from the TIF, display a distinct mesenchymal transcriptional program. The resulting tumor budding signature (with up to 28 distinct genes identified) links conventional pathological assessment with spatially resolved molecular profiling and identifies EGFR-driven invasive cell states with an increased risk of recurrence, further reinforcing the biological relevance of the LE/TIF ecosystem [54]. EMT is increasingly recognized as a spectrum of intermediate cellular states, ranging from partial EMT (pEMT) to complete EMT (cEMT) [55], with increasing plasticity correlating with higher tumor grade and treatment resistance. Multiple signaling pathways converge to activate EMT programs [56], including TGF-β [57], WNT–β-catenin [58], and EGF/EGFR signaling [59].

Spatial analyses further support a continuum of tumor cell states across these compartments. Cells in the intermediate regions exhibit hybrid molecular features, consistent with a gradual transition rather than discrete subgroups [28]. Spatial trajectory and RNA velocity analyses indicate that TC cells progressively acquire LE-like characteristics as they migrate outward, in line with increasing invasive potential. Notably, LE-associated transcriptional programs are highly conserved across patients and even across tumor types, suggesting shared mechanisms of invasion and metastasis, whereas TC-associated programs remain largely tumor-specific. In silico drug screening has identified compounds capable of disrupting TC–LE signaling crosstalk, underscoring the translational potential of spatial omics approaches [28].

Building on epithelial plasticity and state transitions underlying intra-tumoral heterogeneity and EMT, single-cell analyses have refined the characterization of HPV-associated HNSCC beyond established clinical and prognostic differences. HPV^+^ tumors comprise distinct transcriptional subpopulations, including HPV “off” cells that lack detectable viral gene expression while retaining p16 overexpression [7,60]. This phenotype reflects a decoupling between viral oncogene activity and cell-cycle regulation. Functionally, these cells show attenuated cell-cycle dysregulation and partial reactivation of senescence programs, features that may support survival under therapeutic pressure and contribute to tumor recurrence. In parallel, HPV-driven epithelial reprogramming gives rise to keratinocyte populations with noncanonical differentiation trajectories. Among these, HPV-induced differentiation-dissonant epithelial nonconventional (HIDDEN) cells represent a stable compartment throughout tumor progression. These cells exhibit a hybrid transcriptional state combining epithelial lineage markers with aberrant differentiation programs, extending the spectrum of plastic epithelial states observed in HNSCC. Their maintenance depends on the transcription factor ELF3, a central regulator of epithelial plasticity in HPV-associated carcinogenesis [61]. Collectively, these findings highlight how viral oncogenesis shapes intra-tumoral heterogeneity by generating epithelial states with potential clinical relevance.

Tumor cell plasticity also includes dynamic transitions between stem-like states. Cancer stem cells (CSCs) are key drivers of tumor relapse and therapeutic resistance, and single-cell analyses have revealed marked heterogeneity within this compartment. Integration of publicly available datasets has identified distinct CSC states based on stemness scores, differentiation trajectories, and transcriptional regulatory programs [62]. These include naïve stem-like cells with high proliferative capacity and resistance to apoptosis, as well as more differentiated, pro-inflammatory phenotypes. CSCs also express elevated levels of immune checkpoint molecules, such as CD276 and CD47, consistent with an intrinsic capacity for immune evasion. Stem-like states are often enriched at the TIF, linking CSC programs to specific spatial niches [63].

Core pluripotency regulators sustain the CSC phenotype in HNSCC. Transcription factors, including SOX2, POU5F1, BMI1 and NANOG, promote self-renewal and maintain an undifferentiated state [64,65,66]. These factors coordinate transcriptional and epigenetic programs that underpin tumor initiation, plasticity, metastatic potential, metabolic adaptation, and therapeutic resistance [67]. CSC persistence is reinforced by intrinsic resistance mechanisms, including enhanced DNA damage repair, cellular quiescence, and ABC transporter–mediated drug efflux [68,69], together with metabolic reprogramming that enables adaptation to microenvironmental stress [70].

CSCs actively interact with and reshape the tumor microenvironment. Ligand–receptor analyses reveal extensive crosstalk with immune and stromal cells, the extracellular matrix and inflammatory signaling networks. Through these interactions, CSCs promote immune evasion and establish an immunosuppressive niche [71]. Mechanistically, they suppress antitumor immunity via both direct cell–cell interactions and the secretion of immunomodulatory cytokines, such as TGF-β and IL-10, which drive the activation of myeloid-derived suppressor cells (MDSCs) and regulatory T (Treg) cells [72,73]. Collectively, these features position CSC heterogeneity as a key driver of tumor persistence, immune suppression and therapeutic resistance, and as a major determinant of poor prognosis in HNSCC.

## 5. Stromal and Immune Microenvironment: Structure and Cellular Interplay

### 5.1. The Stromal Microenvironment

The stromal microenvironment of HNSCC is a dynamic and functionally heterogeneous ecosystem and is shaped by continuous bidirectional interactions between tumor cells, fibroblasts, and immune cell populations. Within this ecosystem, epithelial plasticity, fibroblast activation, and myeloid-driven immunosuppression converge to sustain tumor progression and therapeutic resistance (Figure 2).

Invasive epithelial cells actively remodel the surrounding stroma, promoting the activation of fibroblasts into cancer-associated fibroblasts (CAFs), which, in turn, enhance the invasion of cancer cells [74,75]. Bulk and single-cell RNA sequencing have identified multiple CAF subpopulations with distinct functional states, including myofibroblastic CAFs (myCAFs), which are associated with extracellular matrix remodeling, and inflammatory CAFs (iCAFs), which produce cytokines and chemokines linked to immunosuppression and, in some cases, are predictive of response to immune checkpoint blockade [76,77]. Spatial transcriptomics has further refined this framework by identifying a CAF subset characterized by high expression of CXCL9, CXCL10, CXCL12, and Galectin-9, associated with immune evasion [78]. Consistently, CAF-related transcriptional signatures highlight extensive extracellular matrix remodeling and collagen organization and are associated with poor prognosis, particularly in HPV-positive HNSCC [79].

Recent ST studies have begun to delineate functional metabolic coupling between tumor and stromal compartments. In oral squamous cell carcinoma, integrative single-cell and spatial analyses identified metabolically distinct niches [29]. Hypermetabolic regions, characterized by increased glycolytic activity and hypoxia-associated signatures, were predominantly localized within tumor cell clusters and associated with immunosuppressive features. These metabolic states reprogram the surrounding stroma. Lactate released by hyperglycolytic tumor cells is taken up by adjacent fibroblasts, inducing their differentiation into iCAFs via HIF1 activation. In turn, iCAFs upregulate CXCL12, which spatially co-localizes with CXCR4^+^ Tregs. These recruited Tregs secrete TGF-β, reinforcing immune suppression and inhibiting cytotoxic T-cell activity. Collectively, these interactions establish a self-sustaining immunosuppressive circuit that supports tumor invasion, immune cell recruitment and metabolic adaptation within the tumor microenvironment. Although these metabolic interactions are primarily inferred from ST signatures, transcriptional programs do not necessarily reflect metabolic flux or local metabolite abundance. The future integration of ST with spatial metabolomics will therefore be essential to resolve metabolic exchanges and validate these inferred stromal–tumor circuits [80].

### 5.2. The Immune Compartment: The Myeloid Cells

ST revealed that immune infiltration in HNSCC is highly compartmentalized, a key determinant of immunotherapy response and clinical outcome. Tumor-associated macrophages (TAMs) represent the dominant myeloid population driving disease progression. TAM enrichment correlates with recurrent and metastatic disease, poor outcome, and elevated PD-L1 expression, and these cells frequently spatially co-localize with CD8^+^ T cells [81]. Recent evidence further supports a direct pro-tumorigenic role of TAMs, showing that SPP1^+^ subsets promote tumor proliferation and migration through NF-κB–dependent production of pro-inflammatory cytokines, such as TNF-α and IL-1β [82]. Single-cell and spatial analyses have further refined TAM heterogeneity, identifying SPP1^+^CCL18^+^ and SPP1^+^FOLR2^+^ subsets enriched at the LE and associated with metastatic transcriptional programs and adverse survival independently of HPV status [83].

In parallel with TAMs, myeloid-derived suppressor cells (MDSCs) represent a major component of the tumor immune microenvironment. MDSCs include polymorphonuclear (PMN-) and monocytic (M-) subsets, each exerting distinct but complementary suppressive functions. PMN-MDSCs inhibit T-cell proliferation through reactive oxygen species and ARG1 activity, whereas M-MDSCs produce nitric oxide, express inducible nitric oxide synthase, and retain the capacity to differentiate into TAMs [84]. Collectively, MDSCs promote tumor progression through Treg activation, Th2 polarization, disruption of arginine and cysteine metabolism, and suppression of natural killer (NK) cell activity [85]. Their marked plasticity and immunosuppressive potential make them attractive therapeutic targets [86]. For example, combined blockade of IL-6 and CCR2 increases NK cell infiltration and activation and improves antitumor efficacy in HPV-negative HNSCC, highlighting a potential strategy to overcome immune evasion [87].

### 5.3. The Immune Compartment: The Lymphoid Cells

The immune microenvironment of HNSCC is highly structured and spatially organized, with distinct niches governing immune activation, immune suppression, and therapeutic response (Figure 2). ST demonstrates that CD8^+^ T cells, the most functionally active immune population, are preferentially enriched at the TIF, an immune “hot” region characterized by high interferon gamma (IFNγ) signaling. By contrast, regulatory T cells (Tregs) accumulate preferentially within the TC and LE, which represent immune “cold” regions enriched in suppressive TGF-β and IL-10 signaling [26,88]. Accordingly, T-cell function in HNSCC is shaped not only by the canonical differentiation pathway but also by spatial context, which regulates T-cell retention, survival, metabolic fitness, and responsiveness to immunotherapy [89]. For instance, chronic antigen exposure, such as that induced by cancer, drives T cells towards an exhausted phenotype, characterized by loss of any immune capacity of response, sustained expression of inhibitory receptors (PD-1, TIM3, LAG-3, and TIGIT) and upregulation of exhaustion transcription factors, like TOX [89].

Immune composition and spatial architecture further diverge according to HPV status. HPV^+^ tumors typically exhibit an inflamed phenotype characterized by abundant T-cell, B-cell, and NK-cell infiltration, together with elevated PD-1 and PD-L1 expression [90,91,92]. scRNA-seq and ST analyses have identified progenitor-like and effector CD8^+^ T-cell states associated with responsiveness to PD-1/PD-L1 blockade [93]. HPV^+^ tumors frequently harbor tertiary lymphoid structures (TLSs), indicative of organized local immune responses. By contrast, HPV^−^ tumors display a highly compartmentalized architecture, in which immunosuppressive cell populations are preferentially concentrated within the TC, thereby limiting effective antitumor immunity [81,94,95]. These differences underscore the profound impact of viral etiology on both the composition and spatial organization of the tumor immune landscape.

B cells represent a key—but often underappreciated—component of the immune microenvironment in HNSCC and are predominantly localized within TLSs, where they interact with CD8^+^ and helper T cells [81]. TLSs are spatially organized immune niches that orchestrate local antitumor immune responses against tumor antigens [96]. TLSs exert effects dictated by their cellular functional state [97]. Mature TLSs, characterized by germinal centers and distinct transcriptional signatures, are associated with robust antitumor immune responses and improved clinical outcomes, largely independent of HPV status and PD-L1 expression [81,97]. Immature TLSs may represent transitional states that can either progress toward fully mature structures or remain functionally inert, depending on local cytokine signaling, stromal interactions, and tumor-intrinsic factors. The balance between mature and immature TLSs may influence response to immunotherapy, with mature TLS-rich tumors showing increased sensitivity to immune checkpoint blockade. Therefore, TLS maturation status is increasingly recognized as a critical determinant of immune competence and a potential biomarker for patient stratification in HNSCC [98,99].

Collectively, these findings support a model in which therapeutic resistance is spatially compartmentalized within protective immune niches enriched in immunosuppressive and drug-resistant cellular states. Spatially resolved omics approaches therefore extend the “hot versus cold tumor” paradigm beyond PD-L1 expression alone and provide a biological framework for biomarker development and patient stratification in immunotherapy-based treatments.

## 6. Translating Omics-Derived Biology into Mechanism-Guided Therapeutic Strategies

Omics-derived prognostic and predictive biomarkers are increasingly enabling biologically refined patient stratification in next-generation clinical trials for HNSCC, supporting the development of rational therapeutic combinations and biomarker-guided treatment strategies. Here, we focus on three major targets of therapy: EMT/stemness phenotype, stromal signaling, and the immune microenvironment. We restrict our analysis to the major trials that have progressed beyond the preclinical stage and are currently under clinical investigation.

Targeting tumor plasticity and its interaction with the tumor microenvironment is emerging as a central strategy to improve treatment efficacy in HNSCC. Pathways controlling EMT, stemness, immune evasion, and stromal remodeling are closely interconnected and shape therapeutic response. Increasing evidence indicates that substantial spatial heterogeneity exists even within individual lesions, limiting the ability of single biopsies to capture clinically relevant tumor and immune diversity. These observations highlight the need for multi-region and longitudinal sampling approaches, particularly when biomarker-driven therapeutic stratification is pursued.

Among the pathways implicated in these adaptive programs, TGF-β signaling has a central role. Therapeutic strategies targeting this axis, including bifunctional agents designed to simultaneously modulate immune checkpoints and TGF-β activity, have entered clinical evaluation and show manageable safety in HNSCC [100]. Bintrafusp alfa, a bifunctional fusion protein targeting both PD-L1 and TGF-β, has demonstrated clinical activity across PD-L1 subgroups, including heavily pretreated HPV^+^ patients [101]. Encouraging activity has also been reported in the neoadjuvant setting in combination with a tumor vaccine, with evidence of pathological downstaging and improved recurrence-free survival [102]. Similarly, ficerafusp alfa, a bifunctional EGFR–TGF-β-targeting antibody, is currently under investigation in the FORTIFI-HN01 trial in R/M HNSCC, with the aim of overcoming TGF-β–mediated immune exclusion [103]. In line with this rationale, combined inhibition of EGFR and c-MET signaling has also shown promising activity, supported by phase II evidence demonstrating improved progression-free survival with HGF inhibition in combination with EGFR blockade [103].

Transcriptomic analyses have identified key pathways in poorly differentiated and highly plastic tumor cells that can be targeted therapeutically. These include activation of MAPK/ERK, PI3K–AKT–mTOR and angiogenic programs, which are linked to therapeutic resistance [28]. These states are also characterized by high proliferative activity, supporting the rationale for targeting cell cycle regulators. Preclinical studies demonstrated that CDK4/6 inhibition reduces tumor cell viability in HPV-negative HNSCC models [104], and a phase II window-of-opportunity trial is currently evaluating abemaciclib with or without nivolumab in HPV-negative HNSCC to assess pharmacodynamic modulation of the microenvironment prior to surgery. In parallel, targeting signaling pathways that sustain these transcriptional programs, such as PI3K/mTOR, has shown early clinical activity in molecularly defined subgroups, such as patients with NOTCH1-mutant tumors [105,106].

Immune modulation strategies aim to convert “immune-cold” tumors into “immune-hot” tumors that are more responsive to immune checkpoint blockade. Activation of co-stimulatory pathways, such as CD40, has shown preliminary clinical activity in combination with pembrolizumab [107]. In parallel, inhibition of alternative immune checkpoints, including TIGIT and LAG-3, is emerging as a promising strategy to restore dysfunctional T cell states and enhance responsiveness to immune checkpoint blockade [108]. Consistently, the combination of vibostolimab (anti-TIGIT) with pembrolizumab demonstrated promising antitumor activity in patients with advanced HNSCC and PD-L1 Combined Positive Score (CPS) ≥ 1 in the phase II KEYVIBE-005 study [109].

LAG-3, another inhibitory receptor implicated in T cell exhaustion, has also emerged as a relevant target. Its blockade can restore T cell function and enhance antitumor immunity [110,111]. Early clinical data with fianlimab (anti–LAG-3) plus cemiplimab (anti–PD-1) in R/M HNSCC have shown durable responses and a safety profile, supporting further investigation of dual checkpoint inhibition strategies [112]. Nevertheless, durable benefit from these approaches remains restricted to a subset of patients, emphasizing the need for biomarkers capable of distinguishing constitutive from adaptive immune exclusion states.

Developmental and stemness-associated pathways may further contribute to immune suppression in HNSCC. Aberrant Sonic Hedgehog (SHH) signaling promotes an immunosuppressive tumor microenvironment [113], being positively associated with naïve macrophages and resting memory CD4^+^ T cells, together with reduced infiltration by activated CD4^+^ and CD8^+^ T cells. These observations suggest that SHH signaling contributes not only to stemness maintenance but also to immune evasion. Accordingly, SHH inhibition may enhance intratumoral recruitment of cytotoxic and antigen-presenting immune populations [114]. On this basis, a phase I study evaluated sequential pulse dosing of sonidegib, an oral inhibitor targeting the Hedgehog pathway, followed by pembrolizumab in advanced solid tumors, including HNSCC [115]. Although preliminary, these studies illustrate how targeting developmental signaling pathways may simultaneously affect tumor cell plasticity and immune response.

An additional example of biologically informed therapeutic stratification is provided by the umbrella trial conducted by the Korean Cancer Study Group in platinum-refractory R/M HNSCC [116]. In this study, involving more than 200 patients, targeted next-generation sequencing (NGS) guided treatment allocation to matched targeted agents, including alpelisib (PIK3CA inhibitor), poziotinib (EGFR/HER2 inhibitor), nintedanib (FGFR inhibitor), and abemaciclib (CDK4/6 inhibitor), with the option of immune checkpoint inhibition (durvalumab ± tremelimumab) in the absence of actionable alterations or upon progression. Promising outcomes in terms of overall response rate, disease-free survival, overall survival, and safety profiles support the feasibility and clinical applicability of NGS-based genomic stratification. These studies exemplify the ongoing transition from empiric treatment paradigms toward new therapeutic stratification strategies, integrating tumor cell states, stromal interactions, and immune context. Table 1 provides a list of representative clinical studies.

**Table 1:** Emerging therapeutic strategies in early-phase controlled clinical trials.

Clinical Study Agent	Target Pathway	Clinical Outcome	Clinical Setting Phase Trial	Reference
Bintrafusp alfa/Tri-Ad5 vaccine	Dual PD-L1/TGF-β axis	Pathological downstaging: 33.3% 2-year RFS: 83.3%	r-LAD Phase I/II	[102] NCT04247282
Ficerafusp alfa/pembrolizumab (FORTIFI-HN01)	Dual EGFR/TGF-β axis	Ongoing trial	R/M HPV- Phase II/III	[103] NCT06788990
Ficlatuzumab ± cetuximab (phase II)	Dual EGFR/HGF-c-MET pathway	Met primary endpoint (median PFS); supports phase III evaluation	R/M Randomized, noncomparative phase II	[117] NCT03422536
Abemaciclib	CDK4/6	Ongoing trial	r-LAD HPV- Single-arm window trial Phase II	University of Arizona NCT04169074
Bimiralisib	PI3K/mTOR/NOTCH1-mutant	ORR: 17% PFS: 5 months OS: 7 months	R/M Open-label, Single Arm Two-stage	[106] NCT03740100
CDX-1140/pembrolizumab	CD40 agonist /PD-1	Preliminary activity in PD-1-refractory HNSCC	Advanced solid tumors, including HNSCC Phase I	[107] NCT03329950
Vibostolimab + pembrolizumab (KEYVIBE-005)	TIGIT/PD-1	ORR: 29% PFS: 4.1 months OS: 15.5 months	R/M Phase II	[109] NCT05007106
Fianlimab/cemiplimab	LAG-3/PD-1	ORR: 33–7% PFS: 2.0–4.1 months	R/M Two expansion cohorts Phase I	[112] NCT 03005782
Sonidegib/pembrolizumab	Hedgehog pathway/PD1	Preliminary activity in PD-1-refractory HNSCC	R/M Phase I	[115] NCT04007744
KCSG HN 15–16 TRIUMPH Trial	PI3K, EGFR/HER2, FGFR, CDK4/6, immune checkpoints	Improved ORR, DFS, OS: supports NGS-guided stratification	R/M Genomic profile-based umbrella trial single-arm phase II	[116] NCT03292250

RFS: recurrence-free survival; PFS: progression-free survival; ORR: overall response rate; OS: overall survival; DFS: disease-free survival.

## 7. Integrative Clinical Stratification in HNSCC: Advancing the Implementation of Transcriptomics Approaches

Having established that omics may provide a framework for refined HNSCC patient stratification in next-generation clinical trials, and that omics-based therapies have progressed from basic research to clinical application, the key challenge is to strengthen the interplay between basic research and clinical practice.

This transition still presents significant limitations that cannot be overlooked. Despite the high resolution afforded by scRNA-seq and ST, most currently available HNSCC datasets remain constrained by relatively small and clinically heterogeneous patient cohorts [118,119]. Increasing the number of profiled cells should not be conflated with higher statistical power, as limited biological replication may fail to capture the full spectrum of inter-patient variability driven by differences in tobacco and alcohol exposure, comorbidities, prior treatments, HPV status, and anatomical subsite. These limitations are increasingly recognized in both individual studies and recent efforts to integrate single-cell datasets into unified HNSCC atlases.

Beyond clinical heterogeneity, tissue-intrinsic biological variables may further complicate interpretation of the tumor microenvironment, particularly in oral cavity cancers. Chronic inflammations such as periodontitis can reshape baseline immune composition through the expansion of macrophages, myeloid-derived suppressor cells, and dysfunctional or exhausted T-cell populations, thereby generating inflammatory programs that may partially overlap with those induced by the tumor [120]. In addition, conventional protocols for tissue dissociation and poly(A)-based sequencing strategies incompletely capture microbial contamination, biofilm-associated organisms, and other non-polyadenylated transcripts, limiting the ability to resolve host–microbiome interactions. Collectively, these technical and biological confounders can obscure the distinction between pre-existing inflammation and tumor-driven immune response. This highlights the need for larger, clinically stratified, and multimodal spatial datasets to accurately define HNSCC ecosystem architecture.

While acknowledging the limitations outlined above, recent technological advances are increasingly bridging the gap between discovery and clinical application. Importantly, scRNA-seq can now be performed on formalin-fixed, paraffin-embedded (FFPE) tissues, with demonstrated concordance with fresh tissue-based scRNA-seq [121], highlighting the growing robustness and translational readiness of these approaches.

**Figure 3:**
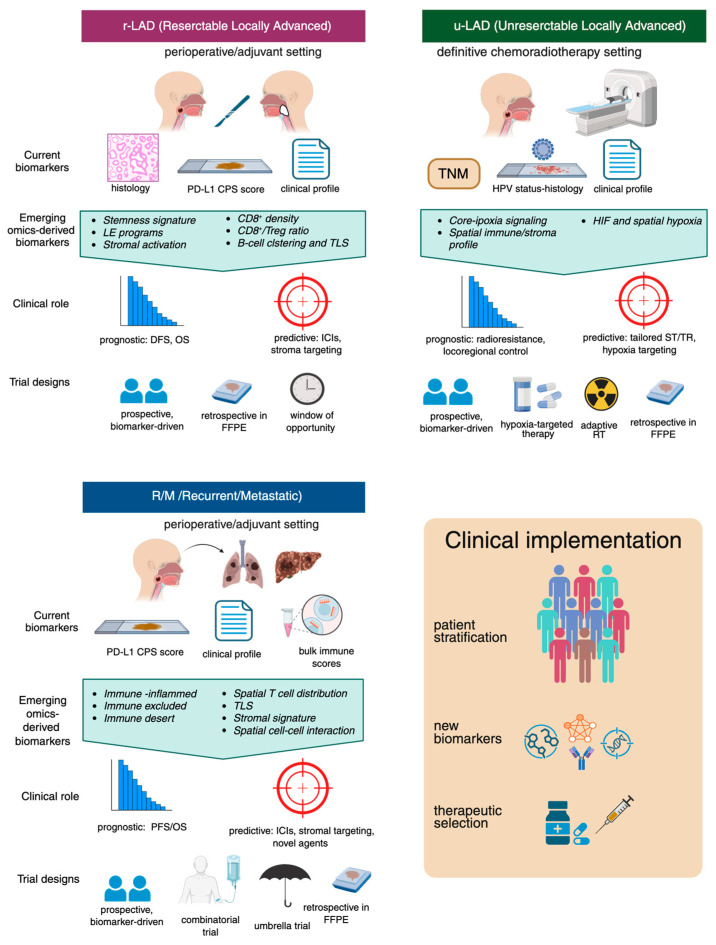
Transcriptomics integration for patient stratification and trial design in HNSCC. Schematic overview of three clinical settings, r-LAD, u-LAD, and R/M HNSCC. Current biomarkers and biomarkers emerging from new omics approaches (scRNA-seq and ST) are shown, together with their clinical role in terms of prognostic and/or predictive markers and the potential clinical trials that may be designed based on these biomarkers in each disease. This figure was created with BioRender.com.

Similarly, ST enables high-resolution spatial mapping of RNA species in routine FFPE specimens, facilitating retrospective analyses of large clinical cohorts [122,123]. Together, these approaches provide a framework for integrating omics-derived biomarkers into both prospective and retrospective study designs. From this perspective, we describe biomarkers identified as potential drivers across the three most clinically challenging HNSCC settings: r-LAD, u-LAD, and R/M disease. Figure 3 summarizes the opportunities offered by scRNA-seq and ST across these settings and outlines potential future trial designs.

In high-risk r-LAD, disease remains surgically resectable but is characterized by aggressive features and early recurrence. Current biomarkers, beyond staging, rely on histopathological assessment, including margin status (close margins), extranodal extension (ENE), PD-L1 CPS, and clinical profile. Standard management consists of adjuvant chemoradiotherapy, as prior immunotherapy-based combinations have failed to improve outcomes [15,16,17,124]. Recently, two landmark phase III trials, NIVOPOSTOP and KEYNOTE-689, demonstrated clinically meaningful benefit from incorporating immune checkpoint inhibitors into treatment [125,126]. Both confirmed safety and represent a significant advance, but key limitations remain, including inconsistent signals in regional control, unresolved mechanistic drivers, a modest pathological response rate (~9%), reduced benefit in patients > 65 years (potential immunosenescence), and limited generalizability (including T4b/N3 disease, HPV-positive tumors, low PD-L1 CPS, and poor performance status). Notably, perioperative immunotherapy appears more effective in selected subgroups, particularly those with high PD-L1 expression, raising concerns about overtreatment in unselected populations [127]. This underscores the need for refined biomarkers beyond clinicopathologic parameters and PD-L1 scoring, including integration of computational pathology with transcriptomic profiling [128,129].

Tumor-intrinsic stemness signatures and invasive LE programs are strongly associated with poor prognosis [28]. In parallel, spatial immune features, including high CD8^+^ T-cell density at the tumor margin, favorable CD8^+^/Treg ratios, B-cell clusters, TLSs, and distinct stromal activation states, provide superior prognostic and predictive resolution compared with conventional immunoscore [130]. Together, these data highlight that therapeutic response is governed by coordinated interactions across tumor, immune, and stromal compartments. Recent evidence further delineates tumor-intrinsic and microenvironmental determinants of immunotherapy response. Malignant programs involving interferon signaling and MHC class II expression are associated with response to ICIs, indicating an active role of tumor cells in shaping immune recognition [131]. In parallel, stromal remodeling during immunochemotherapy, characterized by specialized endothelial venules and distinct CAF subsets, can establish permissive immune niches, whereas alternative stromal states are linked to resistance [132]. Extending these mechanistic insights, Xiang and colleagues [133] demonstrated the clinical benefit of perioperative immunochemotherapy and identified a specific T-cell subpopulation and TLS as correlates of response. These findings support a model in which spatially organized multicellular ecosystems determine treatment response.

Significant unmet needs remain in u-LAD and R/M disease [134,135]. In u-LAD, tumors are not amenable to surgical resection due to extensive local invasion, vascular involvement, or anatomical constraints, and are primarily managed with concurrent chemoradiotherapy. Current biomarkers are limited to TNM stage, performance status, and histology/HPV status. Hypoxia is a well-established driver of radioresistance; however, bulk RNA-seq–derived hypoxia signatures show inconsistent performance, limited gene overlap, and variable prognostic value [136,137]. These signatures are highly heterogeneous and not directly comparable across studies. Integration with scRNA-seq reveals that hypoxia-associated genes are expressed by tumor, stromal, and immune compartments, confounding bulk-based interpretation [138]. This challenges the assumption that hypoxia signatures directly reflect the complex cellular response to oxygen deprivation. Although canonical hypoxia biomarkers such as HIF-1α, GLUT1, and lactate have long been associated with poor prognosis, treatment resistance, and adverse clinical outcomes in oral cancer [139], these markers do not resolve the cellular origin or spatial distribution of hypoxic responses. Single-cell–derived hypoxia signatures therefore represent a conceptual advance by resolving tumor cell–intrinsic hypoxia programs from stromal and immune-associated transcriptional responses. Tumor cell-specific core hypoxia signatures derived through single-cell deconvolution provide a biologically refined alternative to conventional bulk hypoxia scores, supporting their value for clinical stratification and identification of radioresistant tumors. However, hypoxia alone does not capture the spatial and immunological complexity of the tumor microenvironment. Accordingly, integration of hypoxia metrics with spatial immune profiling may provide a more comprehensive stratification framework in u-LAD.

Spatial immune profiling is even more critical in R/M HNSCC. R/M disease, defined by local–regional relapse after definitive therapy or distant dissemination, is generally incurable and managed based on clinical features, PD-L1 CPS, and non-specific immune scores. Immune checkpoint inhibitors, alone or in combination with chemotherapy, represent the standard of care; however, clinical benefit remains limited by primary/acquired resistance and lack of predictive biomarkers beyond PD-L1 CPS [140]. By integrating gene expression with spatial architecture, distinct immune ecotypes have been identified that provide greater predictive value than conventional biomarkers [141]. These include immune-inflamed tumors with cytotoxic T cells and IFNg signaling, as well as immune-excluded and immune-desert phenotypes, in which stromal barriers or immune paucity limit response. Importantly, treatment outcome depends not only on immune cell abundance but also on spatial organization: similar immune densities may result in different responses depending on whether effector cells directly contact tumor cells or remain spatially segregated [141].

Collectively, these findings underscore the central role of spatial immune architecture in shaping immunotherapy response and support its integration into future biomarker frameworks. Prospective validation studies remain warranted and should incorporate complementary strategies, including liquid biopsy. Whereas ST resolves the 3D organization of the tumor microenvironment, liquid biopsy captures its temporal dynamics through circulating biomarkers, such as circulating tumor DNA (ctDNA), circulating tumor cells (CTCs), and salivary components [142,143]. Rather than representing alternative approaches, these modalities interrogate complementary spatial and temporal dimensions of tumor biology and converge within integrated precision oncology workflows for HNSCC.

## 8. Defining the Clinically Actionable Scenario

### 8.1. Single-Cell Atlases as Reference Maps for Clinical Cohorts

From a clinical perspective, scRNA-seq and ST should ultimately inform and standardize decision-making in HNSCC. Rather than remaining primarily descriptive, these molecular and spatial profiling approaches are expected to converge into clinically actionable frameworks.

Three applications appear particularly relevant in HNSCC, as illustrated in Figure 3. First, spatially resolved profiling may refine surgical risk assessment by identifying pEMT programs and invasive phenotypes beyond histologically negative margins, thereby improving postoperative risk stratification and guiding treatment decisions. Second, ecosystem-based biomarkers derived from pretreatment biopsies may support organ preservation strategies by distinguishing radiosensitive, immune-inflamed tumors from biologically aggressive, stromal-dominant ecosystems associated with treatment resistance, thus informing the choice between organ-preserving approaches and radical surgery. Third, spatial characterization of immune architecture may improve patient selection for immunotherapy by differentiating tumors with effective intratumoral T-cell infiltration from those exhibiting immune exclusion, enabling strategies aimed at overcoming microenvironmental resistance. Although these applications remain largely investigational, they highlight how the integration of ST, computational pathology, and artificial intelligence may transform complex tumor ecosystems into clinically actionable decision-support tools.

Prognostic and predictive biomarkers in HNSCC cannot be reliably derived from bulk transcriptomic approaches alone, as these obscure the cellular heterogeneity and spatial architecture of the tumor microenvironment. Single-cell RNA sequencing and spatial transcriptomics have fundamentally expanded our understanding of tumor biology, demonstrating that clinically relevant signals are inherently compartment- and context-specific rather than tissue-averaged. However, translation into routine clinical workflows remains limited by computational, infrastructural, and methodological barriers. A key objective is therefore the development of reference atlases to interpret larger, clinically annotated cohorts.

In this framework, computational deconvolution of bulk transcriptomics represents a key application, enabling insights from small discovery cohorts to be projected onto large clinical datasets, thereby supporting biomarker validation and risk stratification. In parallel, artificial intelligence-assisted digital pathology may bridge molecular spatial maps with routine hematoxylin-and-eosin (H&E) histopathology, providing a scalable interface between omics-based discovery and clinical practice. Machine learning approaches may further link cellular ecosystems to clinically actionable “spatiotypes” (e.g., immune-activated vs. immune-excluded), reducing high-dimensional molecular data into interpretable functional signatures.

### 8.2. Pipeline Standardization and Computational Workflow

Clinical translation of single-cell approaches will require reproducible and standardized analytical infrastructures. Containerized, controlled workflows are essential to ensure portability, reproducibility, and diagnostic consistency across institutions. Benchmarking strategies and scalable frameworks will be critical to address sparsity, batch effects, and multimodal data integration. Emerging large-scale models, federated learning, and multimodal AI architectures may help transform single-cell analysis from a “needle-in-a-haystack” problem into robust decision-support systems. Integration of multimodal transcriptomic and spatial data in prospective clinical studies will be essential for robust biomarker development. Ultimately, progress will depend on rigorous prospective validation, integration into adaptive clinical trial designs, and coupling with computational pathology and artificial intelligence-based decision support systems.

## Data Availability

No new data were created or analyzed in this study.

## Abbreviations

HNSCC	head and neck squamous cell carcinoma
scRNA-seq	single-cell RNA sequencing
ST	spatial transcriptomics
TC	tumor core
TIF	tumor invasion front
LE	leading edge
r-LAD	resectable locally advanced disease
u-LAD	unresectable locally advanced disease
R/M	recurrent/metastatic
HPV	human papillomavirus
PD-L1	programmed death-ligand 1
HIDDEN	HPV-induced differentiation-dissonant epithelial nonconventional
CSCs	cancer stem cells
ABC	ATP-binding cassette
MDSCs	myeloid-derived suppressor cells
Treg	regulatory T
EMT	epithelial–mesenchymal transition
pEMT	partial EMT
cEMT	complete EMT
CAFs	cancer-associated fibroblasts
myCAFs	myofibroblast CAFs
iCAFs	inflammatory CAF
TAMs	tumor-associated macrophages
PMN-MDSCs	polymorphonuclear myeloid-derived suppressor cells
M-MDSCs	monocytic myeloid-derived suppressor cells
TLS	tertiary lymphoid structures
FFPE	formalin-fixed paraffin-embedded
ICIs	immune checkpoint inhibitors
